# Hydroponic potato production in wood fiber for food security

**DOI:** 10.1038/s41538-023-00200-7

**Published:** 2023-06-03

**Authors:** Krzysztof Kusnierek, Pia Heltoft, Per Jarle Møllerhagen, Tomasz Woznicki

**Affiliations:** 1 Department of Agricultural Technology, Center for Precision Agriculture, Norwegian Institute of Bioeconomy Research, Kapp, Norway; 2 Department of Horticulture, Norwegian Institute of Bioeconomy Research, Kapp, Norway

**Keywords:** Agriculture, Developing world

## Abstract

The resilience of global food security is a critical concern. Facing limited access to land and potential disruption of the food markets, alternative, scalable, and efficient production systems are needed as a complementary buffer for maintenance of food production integrity. The purpose of this study was to introduce an alternative hydroponic potato growing system where potatoes are grown in bare wood fiber as a growing medium. A system utilizing drip irrigation and plastic bags as containers was tested for three different types of wood fiber, two cultivars and two fertigation strategies. Implementation of the system resulted in ~300% higher tuber production when compared to the local conventional farming. Mineral composition of the tubers obtained from hydroponic system was similar to the composition of tubers grown in the field and revealed potential for biofortification. In addition, a fertigation strategy where the two application points were separated across the root zone resulted in tubers with dry matter content comparable to the potatoes grown in soil. The recyclability, reusability, and simplicity of this solution may encourage its application for improving security of food production in selected areas of the world as well as its utilization in urban agriculture.

## Introduction

In the coming decades, the expanding population and market volatility will require improvements to the global food system. The food system is not sustainably balanced and does not provide sufficient nutritious food to the world’s population^1^. According to the Food and Agriculture Organization of the United Nations, by 2050, an expected global population of 9.7 billion people will require 70% more food than is consumed today, and 100% more in the developing countries^2^. To secure sufficient food supply for consumers worldwide, increasingly more areas have been currently converted into agricultural land, often at the expense of damaging natural habitats of high biodiversity. It is questionable whether converting more land to produce more food contributes to food security^3^. Godfray and Garnett^4^ pinpointed that the objective of increasing food production needs to be constrained by other, equally important goals for maintenance of sustainability and balance. Food production should be sustainably intensified, i.e., achieved with less impact on the degrading ecosystems, changing climate and decreasing land and water resources. Environmental concerns are, on the other hand, challenging the food security of the rapidly growing population.

Unprecedented urbanization rate alters food systems globally^5^, particularly in sub-Saharan Africa and South Asia^6^. Urbanization triggers changes in food demand towards more nutritious and processed products, converts agricultural land into residential or industrial areas, and forms more complex market linkages^6^. With limited access to land used for agriculture in urban areas, and with higher dependency on transport and infrastructure, the food is either produced more intensively in the remaining agricultural land^7^ or is produced in urban and peri-urban areas on land not classified as agricultural. In fact, urban and peri-urban agriculture has a significant role in food and nutrition security for hundreds of millions of urban dwellers in most low-income nations^8^, although in many cities it has become difficult to get access to the land needed for agriculture^9^. Therefore, there is a need for introducing alternative growing systems and technological solutions to produce food in areas with limited land resources.

Potato is the third most important food crop in terms of global consumption, and it has been highly recommended by FAO as a food security crop while the world is facing challenges of a growing population and disturbances of food supply^10,11^. According to FAO, potatoes bring more yield per unit of cropland in less time than any other major crop^2^. Despite constantly decreasing global potato production area, in 2020, over 360 million tons were produced worldwide, showing a substantial increase from 329 million tons in 2010^12^. Millions of farmers depend on potatoes for both their food and cash income. Meanwhile, unlike the main cereal commodities, it is absent in major international commodity exchanges, meaning that its supply is not affected by the speculative market activities. Potato is one of the global crops with the most diverse distribution pattern^13^. It has been shown that cultivation of potato (and sweet potato) helped to intensify and diversify local food systems otherwise dominated by cereals, as in Asia, helping to strengthen their ability to withstand and recover from crisis^14^. In their recent opinion piece drawn from the situation on the food market during COVID-19 pandemic, Heck et al.^15^ indicated that agricultural innovations should be focused on meeting the needs of the poor and that utilizing biofortified potato and sweet potato would improve nutrition and livelihoods during such crises.

Although potatoes, originating from the high-altitude regions of South America, can be produced under challenging growing conditions, their yield and quality are sensitive to both excess and deficit soil water^16^. Moreover, due to the ongoing and predicted climatic changes the most significant losses in suitability of land for potato production will occur in southern Africa, India and tropical highlands^17,18^. Therefore, it is urgent to explore alternative production systems for maintaining global food security under the future emergency scenarios including expanding areas for potato production, utilizing land with unsuitable climate or degraded and polluted soils.

Hydroponics is a soilless cultivation method in which plants are grown using a nutrient solution. This production system removes the dependency on agricultural land and soil, reduces the presence of diseases and can mitigate the negative effects of extreme weather events utilizing precisely dosed nutrient solution (fertigation). Use of drip fertigation can also significantly decrease N leaching losses because of decreased fertilizer and irrigation needs^19^. Recently, aeroponics, a type of production system where nutrient solution is provided into rootzone in form of aerosol, has been utilized for production of seed potatoes^20^. The aerosol-based fertigation of potatoes has also been investigated by NASA as a proposed strategy for life support systems in extraterrestrial bases^21^ Unlike aeroponics, where the tubers and roots of the potato plant are hanging from a support zone, in hydroponics, growing media, or “substrates” are providing optimal root environment, which ensures an adequate aeration, water, and nutrient supply, making the cultivation less complicated. Traditionally, soilless hydroponic cultivation utilizes peat or coconut coir. Wood fiber produced from softwood tree species is an alternative, renewable and recyclable raw material with lower carbon footprint than peat or coir^22^. This material has also been subjected to initial feasibility test as a potential medium for potato growth and showed promising results^23^.

The purpose of the current work is to propose an alternative hydroponic potato growing system, where potatoes are grown in bare wood fiber. This system is not dependent on agricultural land and has a potential for reducing water losses, therefore it could be considered by policymakers as a tool for improving food in areas with limited land resources or adopted by urban agriculture practitioners. A research hypothesis stating that the quality of potatoes produced hydroponically are different from the conventional production in soil has been tested. Technical details of the system are described for its easy replication while several challenges and opportunities related to this type of production are described for potential future improvements.

## Results

### Yield

**Fig. 1:**
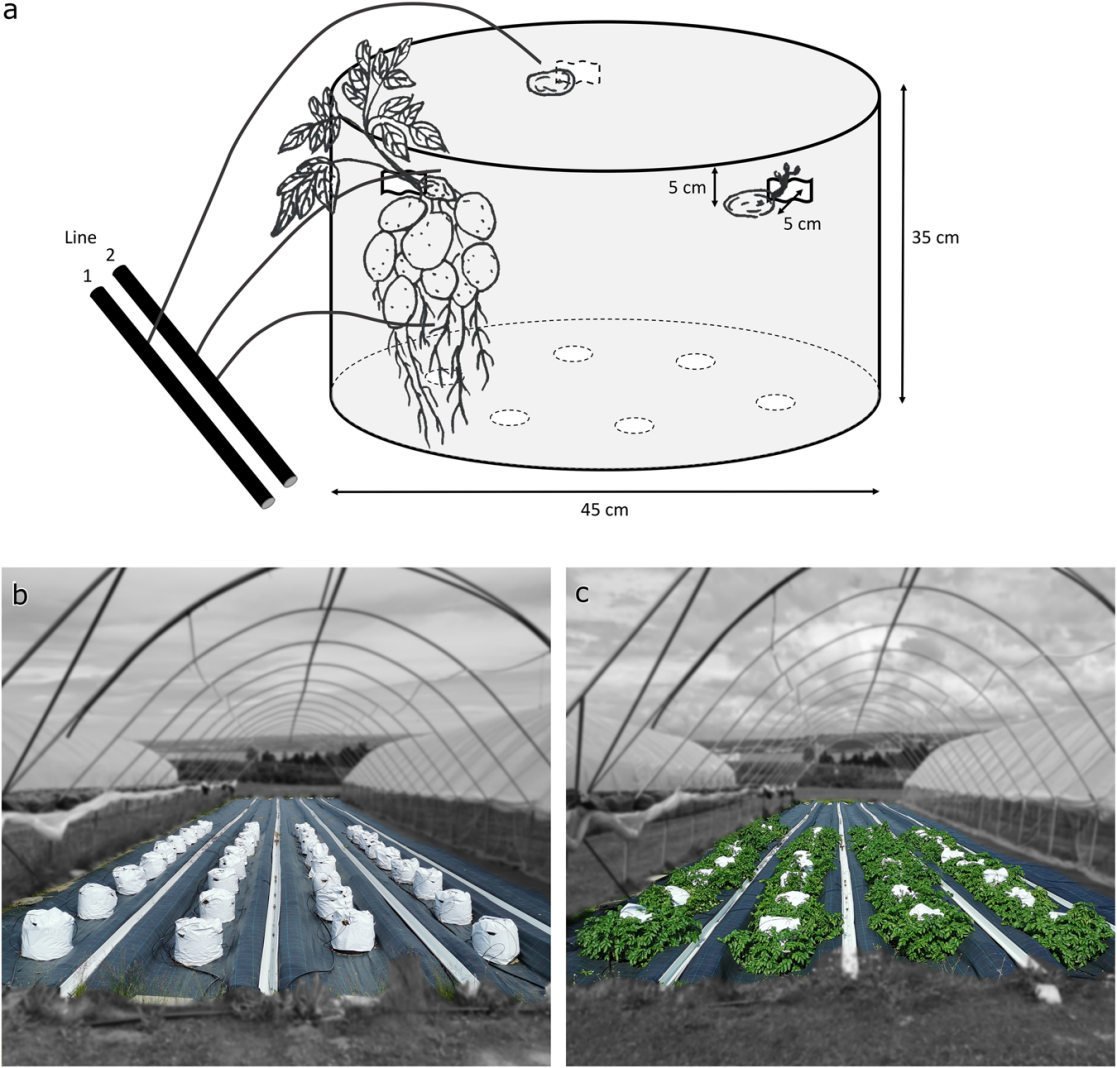
Outline of the growing system unit and the layout of the hydroponic experiment. The outline of the growing system unit (**a**) is not a full representation of the unit, but it is simplified for clearer visualization of its components. The photographs of the layout of the hydroponic experiment were taken (**b**) after planting and (**c**) before harvest.

**Fig. 2:**
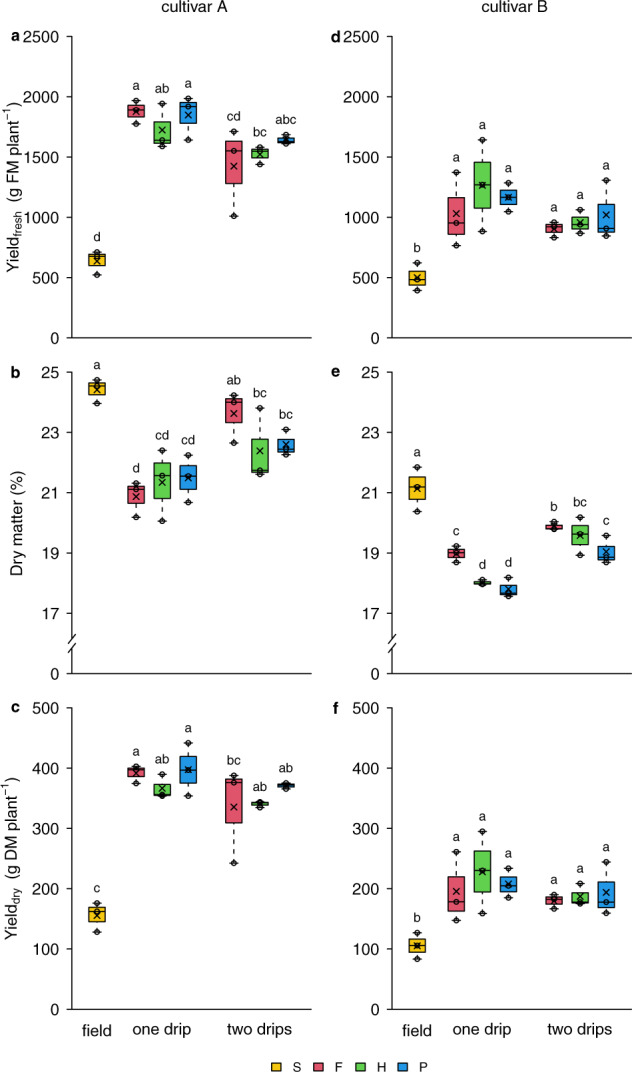
Yield response of the two tested cultivars across production methods and tested growing media. Yield of fresh matter (panels: **a**, **d**), dry matter content (**b**, **e**) and yield of dry matter (**c**, **f**) is presented per plant (*n* = 3) for two distinct potato cultivars: celandine (**a**–**c**; cultivar A) and numbered cultivar (**d**–**f**; cultivar B). Production methods included field control in soil (S) as well as one drip and two drip hydroponic system (see Methods section for details) in three wood fiber substrates: Florentaise (F), Hunton (H) and Pindstrup (P); *n* represents the sample size, and different lower-case letters on the box plots represent significant differences between the treatments at 5% significance level. The vertical lines on the box plots indicate variability outside the upper and lower quartiles, and any point outside those lines is considered an outlier. The symbol × on the boxes indicates the sample mean; the small circles represent individual data points.

The hydroponic growing system (Fig. 1), described in detail in the methods section, unleashed the yield potential of the cultivars tested in the experimental site and produced up to 300% more fresh weight than the field reference for Celandine cultivar (cv.A) and up to 200% for the bred numbered cultivar (cv.B; Fig. 2a, d). Among the two tested fertigation strategies the method delivering all the nutrient solution in the top on the container using a single drip yielded 30% higher fresh matter yield than the methods providing water and nutrients at two levels using two drips. The latter methods, however, produced tubers with significantly higher dry matter content (Fig. 2b, e). This translated to the fact that in terms of dry matter yield, the two tested fertigation methods were quite similar (Fig. 2c, f). No significant difference among the three tested substrates was detected, however a considerable intra-treatment variation should be noted. Both yield and dry matter content of cv.B was significantly lower in comparison to cv.A suggesting higher market potential of the latter.

### Mineral composition

**Fig. 3:**
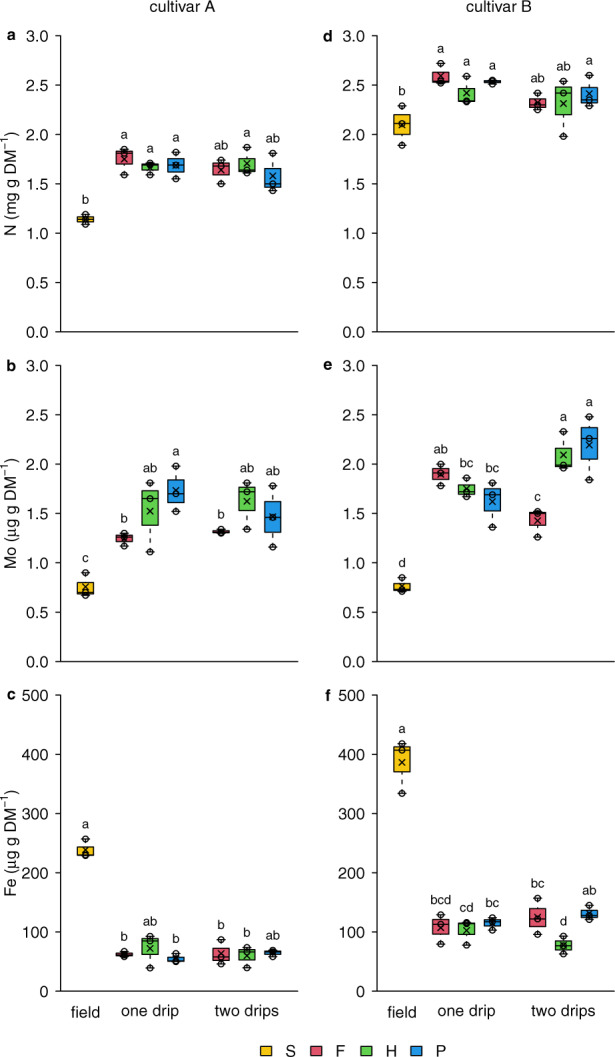
Selected tuber mineral composition for two tested cultivars produced in field and various hydroponic setups. Content of nitrogen (N; panels: **a**, **d**), molybdenum (Mo; **b**, **e**) and iron (Fe; **c**, **f**) in dry matter is presented for two distinct potato cultivars: celandine (**a**–**c**; cultivar A) and numbered cultivar (**d**–**f**; cultivar B). Production methods included field control in soil (S) as well as one drip and two drip hydroponic system (see Methods section for details) in three wood fiber substrates: Florentaise (F), Hunton (H) and Pindstrup (P); *n* represents the sample size, and different lower-case letters on the box plots represent significant differences between the treatments at 5% significance level. The vertical lines on the box plots indicate variability outside the upper and lower quartiles, and any point outside those lines is considered an outlier. The symbol × on the boxes indicates the sample mean; the small circles represent individual data points. See Supplementary Fig. 1 for more details on mineral composition of the tubers.

Dry matter content of the tubers was relatively lower in the hydroponic system as the tubers were less mature at harvest and received higher nitrogen doses as those grown in the field. This difference was also reflected in the mineral composition of the tubers (Fig. 3, Supplementary Fig. 1). Nitrogen and phosphorous content were lower in more mature tubers from conventional production, whereas potassium content was similar in both systems. While nitrogen content did not differ among the two irrigation methods, phosphorous and potassium content did, reaching higher levels at one-drip irrigation treatment. Again, differences among three tested wood fiber types were not significant, with a tendency of Florentaise substrate (F) to support delivering slightly more macronutrients than the two other substrates (Hunton substrate – H, and Pindstrup substrate – P).

Most of the micronutrients were at a similar level in both hydroponic system and in the field control (Supplementary Fig. 1). The two interesting exceptions were molybdenum, much higher in tubers produced in wood fiber, and iron, higher in the tubers grown in the field (Fig. 3). More water was passing through the tuber zone in one-drip treatment, leading to increased nutrient accumulation in tubers produced in this method. Differences between the substrates are not significant. However, a slightly higher nutrient uptake lead to higher dry matter accumulation in F fiber substrate.

### Yield structure

**Fig. 4:**
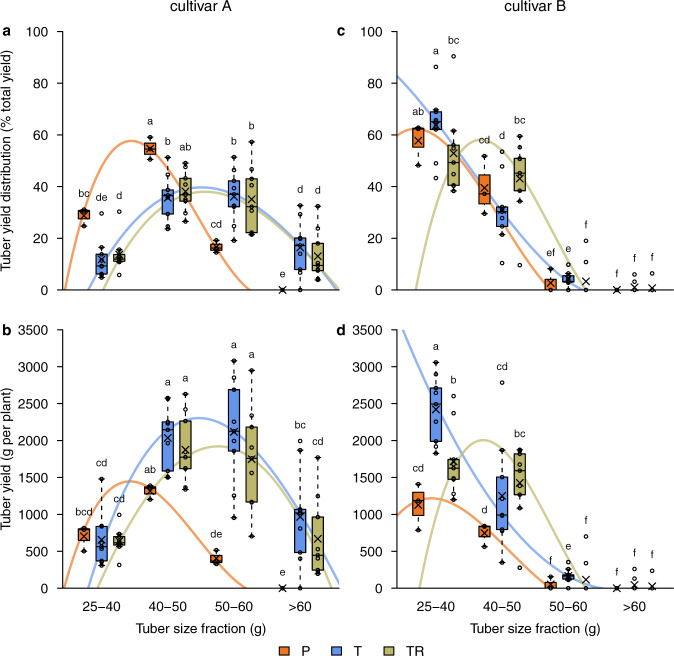
Tuber yield structure across four tuber size fractions of two tested cultivars across the production methods. Tuber yield distribution (panels: **a**, **c**) presented in % of total yield, and tuber yield (**b**, **d**) presented in g per plant (*n* = 3) in two distinct potato cultivars: celandine (**a**, **b**; cultivar A) and numbered cultivar (**c**, **d**; cultivar B). Production methods included natural water delivery in field control by precipitation (P) as well as one drip and two drip hydroponic system deployed in only tuber zone (T) and in both tuber- and root zone (TR), respectively (see Methods section for details); *n* represents the sample size, and different lower-case letters on the box plots represent significant differences between the treatments at 5% significance level. The vertical lines on the box plots indicate variability outside the upper and lower quartiles, and any point outside those lines is considered an outlier. The symbol × on the boxes indicates the sample mean; the small circles represent individual data points. Spline function was enveloped over median values of various treatments to aid visual perception of the results.

There was a clear difference in yield structure tubers grown in hydroponic experiment and in field conditions, with hydroponic system favoring production of the largest tubers (Fig. 4a, b). This difference was clearer for cv.A than for cv.B. In conventional production of cv.A, about 80% of the tubers were classified in the smaller-size categories from 25–50 g, whereas in hydroponic production, the tubers were distributed more equally (Fig. 4a). In cv.B almost all the tubers were classified as small (Fig. 4b). Absence of the largest potatoes (>60 g) was noticed for conventionally cultivated cv.A, while the hydroponic system produced up to 20% of the total yield in this size class, which translated to a substantial portion of the total yield (Fig. 4a).

In cv.A, no real differences in yield structure were observed between the two irrigation methods, while in cv.B the 2-drip treatment produced slightly larger tubers (Fig. 4a, c). Interestingly, there was a difference in yield distribution between the two irrigation methods. One-drip production of cv.A gave higher yields in the two larger fractions than in the two-drip irrigation, accounting for the difference in the total yield between the two methods (Fig. 4b). In cv.B, one-drip irrigation led to high yield of the tubers in the smallest size class, whereas the two-drip irrigation method favored the production of the larger sized tubers (Fig. 4d).

### Parameters influenced by the two irrigation methods

**Fig. 5:**
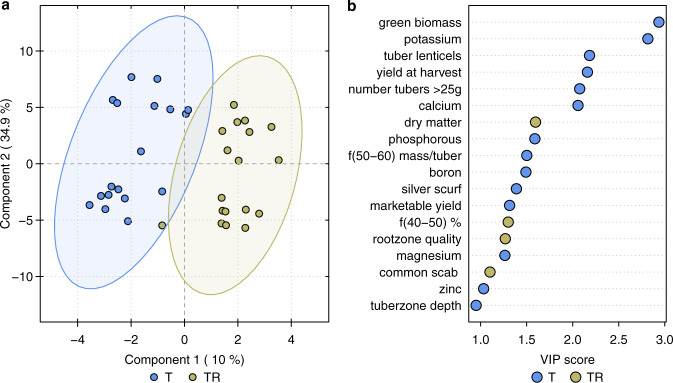
Difference between the two tested fertigation methods and the most influential among the analyzed variables. Difference between the fertigation methods utilizing one drip and two drip hydroponic system deployed in only tuber zone (T) and in both tuber- and root zone (TR), respectively, were analyzed using (**a**) a score plot of Partial Least Squares Discriminant Analysis (PLS-DA). The most influential among the analyzed variables were selected using (**b**) VIP scores calculated from PLS-DA. Higher VIP scores indicated variables with more importance to the model. The color-coding of the scores shows which class had higher values of a given original variable.

All available variables describing the hydroponic experiment were synthesized using a multivariate classification method (PLS-DA, see methods section for details). The results showed a clear difference between the two applied irrigation methods (Fig. 5a). The difference was contained in component 1, which explained 10% of the total model variance and led to the best discrimination of the irrigation treatment groups. Component 2 explained nearly 35% of the variation and was influenced mainly by the difference between the two tested cultivars. Further components, although explaining substantial portion of the variation did not have a large influence on the model as their correlation loadings were under 0.5 and prediction error gradually increased (data not shown). Nonetheless, component 3 was attributed to mineral accumulation in tubers while component 4 to the quality of the substrates.

The analytical method utilized VIP scores and highlighted the most influential variables as affected by the fertigation strategies, indicating which of the two irrigation methods led to a higher level of a particular variable (Fig. 5b). One-drip irrigation method led to higher biomass and mineral accumulation in tubers. Two drip irrigation provided tuber zone condition which was not suitable for excess water accumulation in the tubers. The tubers produced in these conditions had higher dry matter content. In addition, root zone was more dense and penetrated majority of the available growing medium. Less water in the tuber zone led to relatively less swollen lenticels and better disease resistance in the 2-drip system. Silver scurf was the disease that differentiated the two irrigation systems the most, as it is known to be transmitted through the swollen lenticels^24^. One-drip method was more successful in assimilating nutrients in the tubers. Potassium, calcium, boron, magnesium, and zinc were the nutrients that differentiated the two irrigation methods the most. Tuber zone in the bags fertigated with this method was slightly deeper than in the bags fertigated with 2 drips. This analysis showed also that 2-drip irrigation led to higher number of tubers in the consumer-preferred size fraction from 40–50 g, while 1-drip method produced more small-sized tubers (25–40 g).

### Tuber defects and diseases

**Fig. 6:**
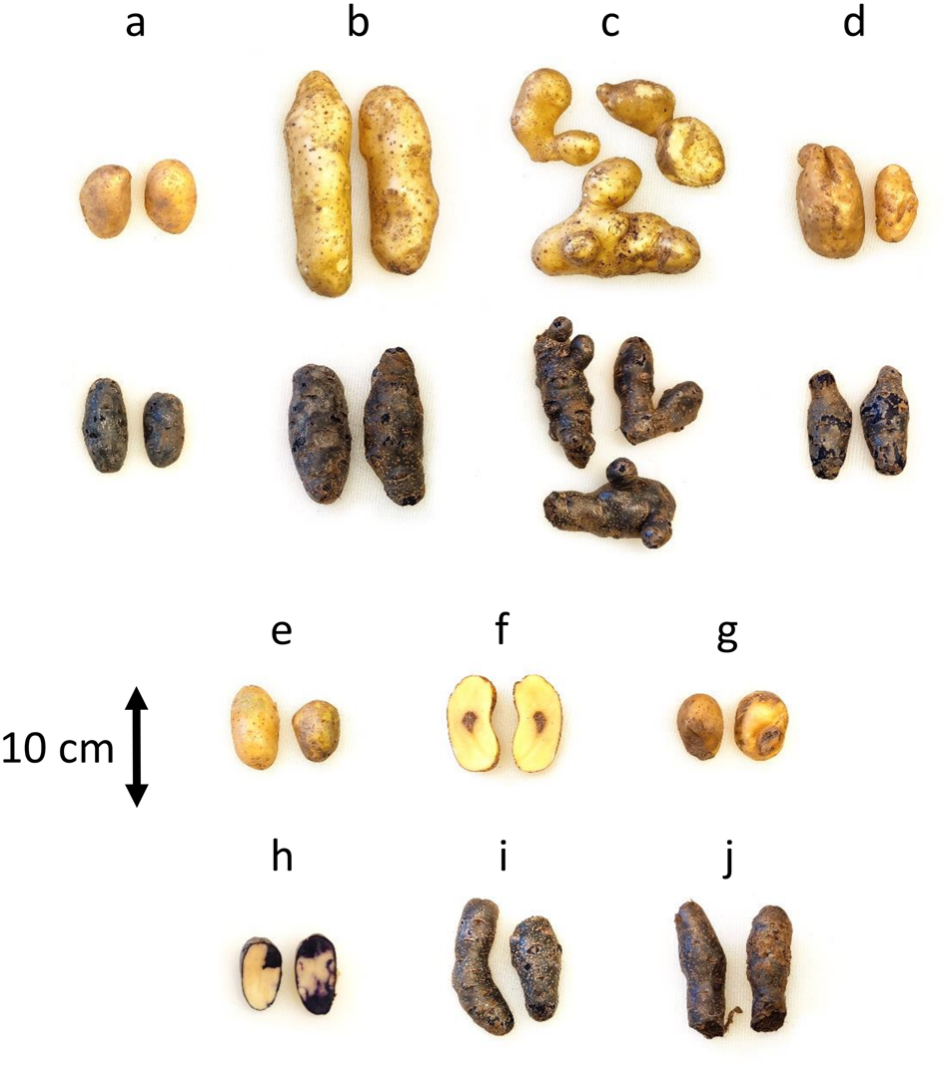
Tuber defects and diseases observed in hydroponic production system. In comparison to (**a**) field control, the observed tuber defects and diseases included: (**b**) large tubers (>60 g), (**c**) deformations, (**d**) cracks, (**e**) greening, (**f**) internal brown spot disease, (**g**) dry rot disease, (**h**) discoloration, (**i**) enlarged lenticels, (**j**) soft rot disease.

The experiment showed that the plants did not utilize the entire space of the container for tuber production, progeny tubers were concentrated only about 20–25 cm around the seed potato. The roots, on the other hand, grew across the whole container and mostly at the bottom. In general, the tubers harvested from hydroponic production in wood-fiber-based substrates were free from quality issues. However, some tubers displayed certain challenges of that system, including occurrence of several types of defects and diseases, which are visually displayed in Fig. 6, and listed in Supplementary Table 1.

In comparison to the field reference (Fig. 6a) some tubers grown in the hydroponic system had a tendency to be large (>60 g), (Fig. 6b), which in average accounted for 16.4% of the total yield (Fig. 4a). In addition, 6% of the tuber yield was found deformed (Fig. 6c, Supplementary Table 1). Relatively high temperature and moisture amplitudes existing in the root environment of the hydroponic system led to incidental formation of growth cracks in some of the tubers (Fig. 6d), but it comprised only 1% of the total yield (Supplementary Table 1). Some tubers (1% on yield basis) of cv.A grew next to the walls of the container, and therefore were partly green during harvest (Fig. 6e, Supplementary Table 1). Some cv.B tubers were registered with purple pigment partly missing (Fig. 6h). Only single tubers of cv.A were infected by dry-conditions diseases, such as internal brown spot disease (Fig. 6f) and dry rot disease (Fig. 6g), see Supplementary Table 1. In cv.B on the other hand, overgrown lenticels (Fig. 6i) and soft rot disease, developed in ca. 5% of the total tuber yield after three months of storage (Fig. 6h, Supplementary Table 1), indicated wet growing conditions.

## Discussion

Hydroponic production of potatoes in wood-fiber-based substrates, as presented here, is a straightforward and scalable solution for mitigating insecurity at the ever-growing food markets, which is gradually deepened by diminishing land resources or acute emergencies. A recent study showed that while the global food demand continues to increase due to population growth and other socio-economic issues, the maximum achievable crop production potential has reached its peak in most regions of the world^25^. As the agricultural land has been utilized nearly to its limits, urbanization has pressed the food supply even further taking over the fertile land^5^. Moreover, the production of sufficient food for the ever-growing population in the available agricultural areas is challenged by the market of non-food crops, such as energy or industrial crops. They often compete with food crops for limiting natural resources, particularly water and land^26^. Meanwhile, the framework of sustainable intensification requires that further exploitation of natural habitats for expansion of agricultural land is limited. This is potentially pushing the new food production sites away from the consumption centers and is challenging an important aspect of food security – food distribution. By the year 2050, 68% of the world’s population is expected to live in cities^2^ as an increasing number of people migrating from the rural areas where they typically lived off the land. In consequence, hundreds of millions of urban dwellers rely on urban agriculture for part of their food consumption or income as they process and distribute their surplus produce^8^. Consequently, urban agriculture is commonly considered as an important contributor to future global food security, which brings the food to the front of the Water-Energy-Food nexus^27^. Therefore, there is an increasing global interest in scaling-up urban agriculture, which is gradually encouraged by prospects of environmental protection, waste management, and energy cost reduction^28^.

Without access to fertile land in urbanized areas, urban farming requires technological innovations such as vertical indoor cultivation or precision agriculture to optimize food production^29^. However, for millions of smallholder farmers, alternative, simple but efficient growing systems with minimal environmental footprint are needed. Accessibility of the system components and materials may be problematic in particular areas, however with support from the local governments, international aid or charity organizations, not only commercial farms, but also small-scale growers can implement simple hydroponic food production.

In general, a closed soilless agricultural production may have benefits over conventional production under certain circumstances. Hydroponic systems are in principle less influenced by the weather conditions which are mitigated by timely dosing of water and fertilizers preventing the negative effects of excess precipitation of drought. This provides good predictability of production. The observed higher Mo concentrations in the hydroponic tubers (Fig. 3b, e) confirmed that using hydroponic systems opens possibilities for straightforward implementation of biofortification strategies of high-calory food, which is in special interest for vulnerable societies^30^.

The hydroponic system designed and tested in this study includes small round plastic-wrapped bags of wood fiber as a growing medium (Fig. 1). Deployment of such a system in areas with relatively high evaporation rates will potentially increase water use efficiency and save water resources. The relatively high porosity of the wood fiber allows air into the root zone and favors high yield formation (Fig. 2). On the other hand, to reach sufficient moisture of the root zone, a high degree of overirrigation is needed. This leads to considerable drainage, which in the further development of the system should be captured and recycled to improve water use efficiency.

An efficient alternative crop growing system has the potential to complement conventional potato production and act as a buffer supporting the resilience of the food market chains. This is especially important as climate change can either reduce or destroy conventionally produced crops. For example, Southern Africa and South America have experienced recent extremes in dry and wet rainy seasons^31^, which have been attributed to global physical mechanisms such as El Niño–Southern Oscillation, sea surface temperature and land–atmosphere feedback^32^. Such changes in temperature and precipitation patterns may trigger extreme weather events that could affect food production^33^.

Traditionally, soilless hydroponic food production utilizes organic substrates like peat and coconut coir for variety of crops, or mineral substrates, as rockwool, which is typically used in professional production of high value crops like tomatoes, peppers and cucumbers. These substrates provide optimal root environment by ensuring adequate aeration, water availability and nutrient supply, however, their production has a high carbon footprint^34^. Wood fiber, on the other hand, the substrate suggested here for potato cultivation, is a renewable and recyclable raw material. It has the lowest carbon footprint among the available raw materials utilized in growing media industry and is increasingly suggested as an important growing medium for plant production^22,34^.

Wood fiber is characterized by the highest porosity among the known organic substrates for hydroponic plant production^22^, which prevents waterlogging and forms the more optimal conditions for root and tuber growth where less precise fertigation strategies are applied. Relatively low bulk density and high porosity of wood fiber favors high oxygen concentration around the tubers that allows fulfilling the yield potential and increase productivity of the potato plants^22^. In a recent study^35^, the authors observed that the mineral composition of the tubers can be affected by the type of production system (conventional vs hydroponic). For example, a higher concentration of P was found in tubers grown in hydroponic production system, which is in agreement with present study. In contrary to other organic media like peat and coir, buffering capacity of wood fiber is low, by which a producer may relatively easily control the pH of the root zone and the uptake of specific nutrients which for example could be utilized for biofortification purpose^30^. Due to this fact, difference in pH may be responsible for varying uptake of macronutrients in the present study and the study of Liszka-Skoczylas et al.^35^. In addition, the wood fiber produced in the defibration process is sterile, therefore, the press from the soil borne diseases is minimized^22^. Moreover, separation of the tubers from the substrate at harvest is straightforward, and the tubers may just be rinsed with running water and be ready for consumption. Interestingly, spent fiber-based growing medium may be used in several growing cycles^36^. Otherwise, the leftover fiber-based substrate can be utilized after plant production as a fuel or soil additive due to the content of the residual salts (nutrients).

Although the highest usability of the system is projected in densely populated tropical areas with limited sustainable wood resources, we do not advocate using local wood species to produce fiber. Softwood that typically grows at high latitudes has proved to be suited for producing fiber for hydroponic production^22^. Normally, after being manufactured wood fiber is relatively dry and can be transported compressed, which lowers their carbon footprint. Alternatively, other biomass constituents, such as miscanthus can be successfully utilized as a growing media^37^. In addition, in urban areas, industrial waste wood (e.g., pallets, untreated wood from furniture industry) processed by hammer mills may be considered as a potential sustainable source of raw materials for growing media production.

The presented production system has a simple low-tech design aimed at straightforward implementation in practice. Supplied with wood fiber, a farmer is required to fill a plastic bag with the growing medium and compress it. Fiber compression and the white color of the plastic on the outside of the bag are suggested for limiting the high temperature and moisture amplitudes.

The seed tubers have been planted in the pockets, made by incisions on the side of the bags, as the original idea was to test the feasibility of placing the bags on top of one another for space-saving vertical cultivation. We noticed a deep seeded tuber challenges the stolon development and reduces the future yield, as some of stolons are bound to grow underneath the plastic bag. In practice it may be needed to manually guide the stolons out from underneath the plastic cover. This system is based on plastic wrapping that should be recycled, however, in various parts of the world this might be challenging. In further development of the system, it is advocated to exchange a simple plastic bag with a reusable tarp or wrapping made of compostable plastic to reduce environmental impact of this type of production.

Although in our experiment we utilized a greenhouse-grade industry standard fertigation system, hydroponic production in wood fiber does not require high investment in equipment. The cheapest diaphragm pump, a venturi valve and a cyclic timer switch may be purchased at the global market for as little as 5$, 5$ and 3$, respectively. The scale of implementation would determine the size of the pump and plumbing. The cost of water hoses and drip valves are estimated to 0.5 $ per bag. According to prof. B.E. Jackson from NCSU, USA, many, if not most wood-based products offer a very competitive or cheaper alternative to traditional substrate materials”^38^. The cost of wood fiber has been estimated in this study at 2$ per growing bag. The amount of fertilizer to feed a single bag of per growing cycle was estimated to ca. 0.2 kg of calcium ammonium nitrate and ca. 0.2 kg of compound fertilizer, which costs approximately less than 1$. In practice, prices of fertilizer and growing media strongly depend on the scale of purchase and fluctuations on local and global market. Power and water supply must be considered additionally.

Regardless of the implementation of the system, it is important to properly place the irrigation drip over the seed potato as the initial growth depends on keeping it moist. Moreover, at a given irrigation level a single drip location favors high moisture of the whole substrate profile and transports all the liquid through the tuber zone. Delivering the same irrigation level into both tuber and root zone limits the growth of aboveground biomass and the amount of water the tubers are directly exposed to, which increased their dry matter (Fig. 2b). While the fresh yield is relatively reduced in respect to one-drip irrigation (Fig. 2a), the dry yield is on relatively similar levels (Fig. 2c). The tested hydroponic system delivered ~300% higher yield than the field reference, which may be attributed to higher root oxygenation and nutrient availability. Stoian et al.^39^ in their study on sweet potato grown in a hydroponic system, also observed that the texture of growing medium improving oxygenation level may positively affect yield formation. In comparison to field reference, relatively higher percentage of large and deformed tubers observed in hydroponic production in wood fiber (Fig. 6, Supplementary Table 1) could be attributed to a not optimal compaction of the fiber in the bags and may be reduced by adjustment of fertigation strategy, higher level of compaction or use of a different texture of growing medium.

In conclusion, the proposed system provides a solution for intensive production of high-quality table potatoes that is able to achieve much higher yield than the field reference with comparable tuber dry matter content and nutritional value (mineral composition). The design allows for easy implementation and scalability of production, even in areas with limited resources. Several challenges of such production have been noted, including the importance of precise placement of seed tubers, longer production cycles due to increased biomass production and a risk of tuber deformation due to increased moisture of the tuber zone. Two different irrigation regimes were suggested, one maximizing the yield and nutrient content, another maximizing dry matter content and minimizing defects of the tubers. In addition, due to character of hydroponic production the system utilizing wood fiber has the potential to precisely adjust pH and macro- and micronutrient composition of tubers and can be used for biofortification.

## Methods

### The hydroponic system and study design

Two potato cultivars were selected for the experiment conducted in NIBIO Apelsvoll Research Station located in SE Norway (60°42N, 10°51 E, 260 m a.s.l.). The first one, Celandine, a potato with a firm texture, was selected as a representative to oval/long formed, tasty cooking potatoes, with high tuber number and good resistance to several diseases, especially common scab. The other cultivar was a late selection (G09-1057) from the Norwegian potato breeding program performed by Graminor (Graminor, Staur, Norway), which was chosen in this study due to its relatively low dry matter potential, unusual shape and color (purple skin and flesh).

A hydroponic system was designed and implemented in this study. Three seed tubers were planted on 3 June 2021 in randomly distributed plastic bags filled with ca. 50 L of wood fibers, forming a cylindrical growing space of 35 cm (about 1.15 ft) in height and 45 cm (about 1.48 ft) in diameter. Seed tubers were placed in relatively deep holes (5–10 cm) made in the top-side section of a bag to ensure even space availability for the new tuber and to prevent them growing against the wall of the bag (Fig. 1a). Each treatment was represented by three replicates. The bags were placed with 80 cm (2.62 ft) between one another (Fig. 1b) ensuring space for expected high green biomass production (Fig. 1c). Three commercial wood fiber variants were tested: Hunton fiber (H) produced from Norway spruce (*Picea abies*), using the defibrator method (Fibergrow, Hunton, Norway), as well as Pindstrup fiber (P) (Forest Gold, Pindstrup, Denmark) and Florentaise fiber (F) (Florentaise Hortipain, France), both produced from Scots pine (*Pinus sylvestris*) using the high-pressure steam method. The nutrient solution was prepared by mixing 1:1 hydroponic quality grade fertilizers Kristalon Scarlet and Calcinit (Yara, Norway). The solution was distributed by drip irrigation providing 1.2 L h^−1^. The hydroponic production was initiated using fertigation solution with electrical conductivity of 1 deciSiemens per meter (dS m^−1^; EC1) three times a day (8:00; 12:00; 16;00) for 7 min intervals. To comply with in-season weather changes and crop biomass growth, from 16 July 2021 the EC of the solution was elevated to 1.8 and it was used to fertigate the plants with intervals of 10 min, four times a day. From 16 August 2021 the EC was reduced to 1.5 and the fertigation regime was split into two treatments; first treatment, with a single drip at the top of the bag (T), and irrigation intervals of 12 min, and the second treatment, with two drips, placed on the top and in the middle of the bag (TR), and fertigation intervals of 6 minutes from each drip. In result, both treatments delivered the same amount of nutrient solution, providing ~10 g N per plant per season. The experiment was finished on 14. September 2021, after a growing season of 104 days and receiving 910.5 growing degree days (GDDs, at base temperature of 7 °C).

Potatoes were also grown in the field for reference. Tubers of both cultivars were planted in the previously prepared field (12 cm depth with a distance of 30 cm within rows and 80 cm between rows), on 31 May 2021, which is a typical planting time in the study area. The field was located in Østre Toten county, one of the main potato production regions in Norway. The field was placed in a fairly flat terrain, on well-drained Endostagnic Cambisol (IUSS Working Group WRB, 2006), with loam and silty sand textures, developed from moraine till deposits, soil organic matter content of 44 g kg^−1^, bulk density of 1.3 Mg m^−3^, and pH measured in water at 6.5. The tubers were randomly harvested from the reference field on 15 September 2021, after 108 days of production, having received 944.7 GDDs. The field was fertilized with a total of 100 kg N ha^−1^ (=833 kg NPK 12-4-18+micro). The field was split-fertilized, receiving 70% of the total dose at planting and the remaining dose 30 days after planting. Mineral fertilizer provided ~2.5 g N per plant per season. The strategy used to control late blight (Phytophtora infestans) and other diseases caused by fungi in the field included the use of Naerstads model to decide the number of applications^40^. Different fungicides (cymoxanil, propamokarp, cyazofamid, mandipropamid and difenoconazole) were used 8 times over the entire growing season, both as stand-alone products and blends, to avoid resistance development. In hydroponic experiment, however, due to the lower disease pressure, only three applications of metalaxyl, mandipropamid and cyazofamid were conducted. In the periods of low precipitation, 4 times during season, the potato field was irrigated using a sprinkler system, receiving ~20 mm per irrigation event.

### Data registration

Both the hydroponic and field experiment were harvested at the same time to ensure straightforward comparison between the two production systems. According to local practice, plant shoot biomass in the field trail was chemically killed, therefore the number of shoots per plant, shoot length and shoot fresh biomass was recorded only for the hydroponic trial where biomass was kept. The quality of the tubers (in terms of overgrown lenticels), green biomass and substrate (in terms of its moisture, rootzone quality and tuber zone depth) was recorded. Unexpectedly, since the yield in the substrate test was extraordinarily high, it was not possible to separate the yield of a single plant. Potato tuber yield was therefore measured per three plants. The tubers were separated into four size fractions, 25–40 mm, 40–50 mm, 50–60 mm and >60 mm, and tubers in each fraction were counted and weighed. The results were then recalculated per single plant. The dry matter content was determined by over- and under-water weight to determine the specific weight of the tubers. The following equation was used to calculate the dry matter content: dry matter = 215.73 * (x − 0.9825), where x is the specific weight calculated as weight in air * (weight in air − weight in water)^−1^. After three months of storage in 4 °C and relative humidity of 90–95%, the classical analysis of tuber quality was performed by trained staff at NIBIO and included visual determination of different potato diseases as described by^24^ as well as registration of weight of the tubers in each sample with selected diseases, i.e.: soft rot, dry rot, brown spot; and defects, i.e.: green tubers, cracks, deformations, discoloration.

Elemental analysis of potato tubers was conducted by the commercial laboratory (Eurofins, Wageningen, Netherlands). NH4, NO3, Cl was analyzed using in- house method, and the contents of macronutrients P, K, Ca, Mg, and S, as well as micronutrients B, Cu, Fe, Mn, and Zn in tubers were analyzed by inductively coupled plasma−optical emission spectroscopy (ICP-OES) using in-house protocols of the laboratory.

### Statistical analysis

Data analysis followed the data presentation paradigm suggested by Weissgerber et al.^41^ and Amrhein et al.^42^. Due to the relatively small dataset, all data points are presented in the figures. Before the analysis, the normality of the data distribution was verified by Anderson-Darling test. For normally distributed data, one-way analysis of variance (ANOVA) was conducted to determine the significance of differences between the groups and Fisher post hoc test was further applied to compare the treatments. For non-normal data distributions, a non-parametric Kruskal-Wallis test was used. The multivariate analysis of all the variables in the study was performed using Partial Least Squares Discriminant Analysis (PLS-DA) to investigate patterns in the data by discriminating groups within the data and to identify the key variables that drive such discrimination, which was performed using the PLS methods called VIP scores. The analyses were conducted using MiniTab statistical software (version 17.2.1, MiniTab, MiniTab Inc., PA, USA) and R Statistical Software (version 4.1.3, R Foundation for Statistical Computing, Vienna, Austria) with RStudio (version 2022.02.2 Build 485, RStudio, RStudio Inc. Boston, USA).

### Reporting summary

Further information on research design is available in the Nature Portfolio Reporting Summary linked to this article.

## Supplementary information

Supplementary information

Reporting summary checklist

## Data Availability

The raw data supporting the findings reported in this study are available on request from the corresponding author.
