# Supplementary material for: Hydroponic potato production in wood fiber for food security
Source: NPJ Sci Food. 2023 Jun 3;7:24. doi: 10.1038/s41538-023-00200-7 (PMC10238232; doi:10.1038/s41538-023-00200-7)
Supplement: Supplementary file 1 — Supplementary information [file 41538_2023_200_MOESM1_ESM.pdf]

Supplementary Information

# "Hydroponic potato production in wood fiber for food security".

Krzysztof Kusnierek, Pia Heltoft, Per Jarle Møllerhagen, and Tomasz Woznicki (NIBIO, Norway)

npj Science of Food

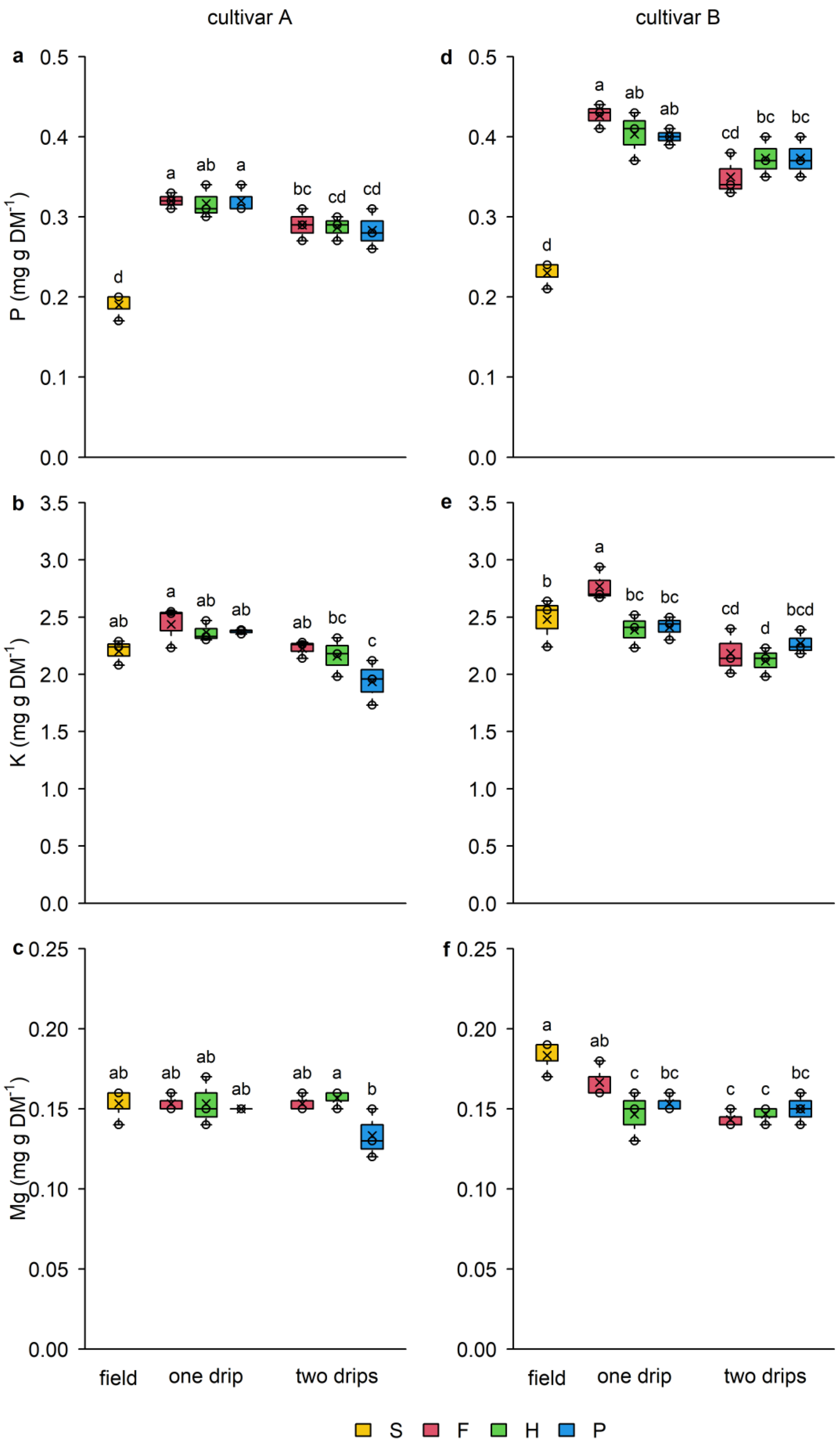

cultivar A
cultivar B
a
b
c
d
e
f
P (mg g DM$^{-1}$)
K (mg g DM$^{-1}$)
Mg (mg g DM$^{-1}$)
field
one drip
two drips
S
F
H
P

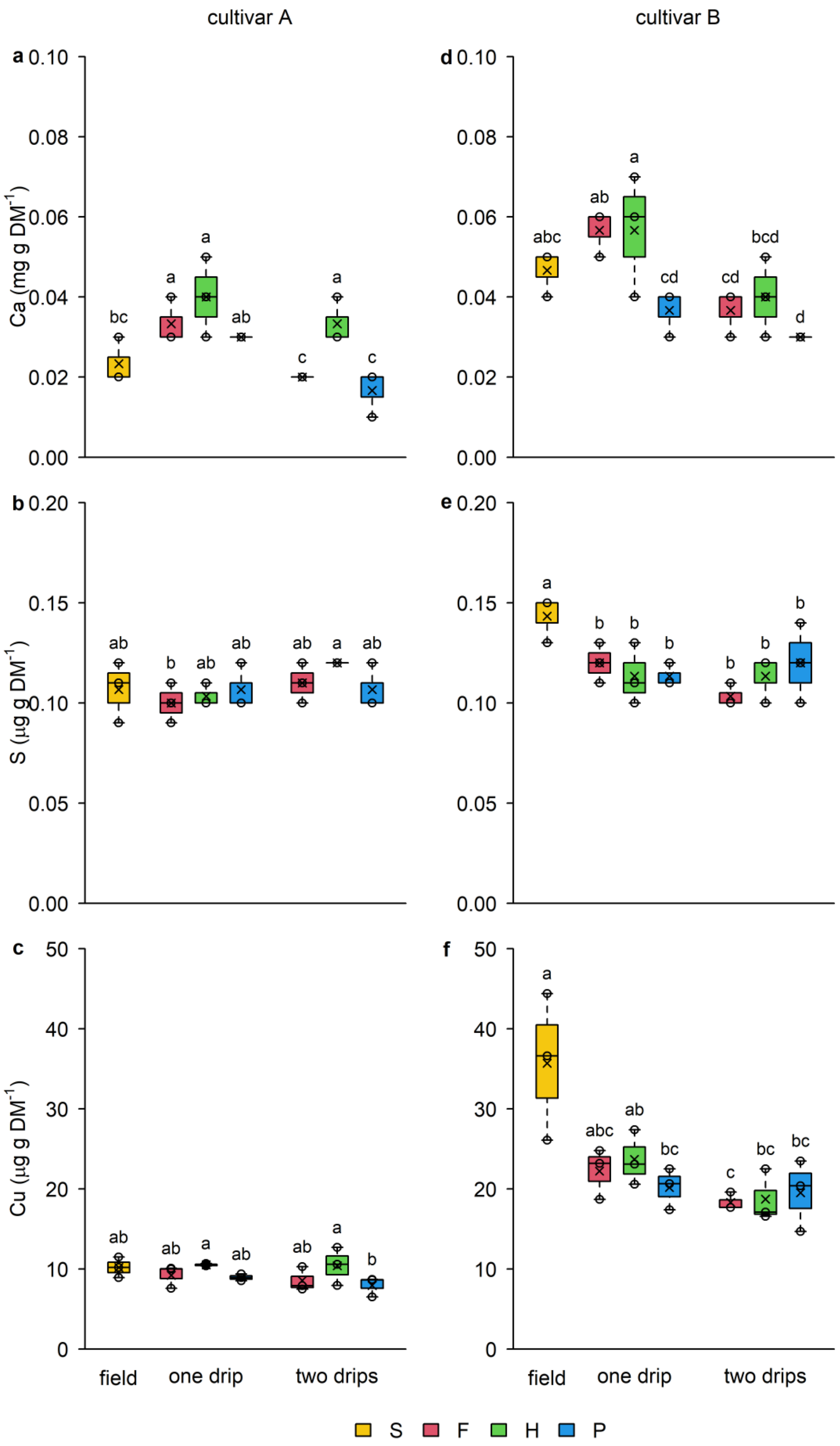

cultivar A
cultivar B
a
d
b
e
c
f
Ca (mg g DM$^{-1}$)
S (µg g DM$^{-1}$)
Cu (µg g DM$^{-1}$)
0.10
0.08
0.06
0.04
0.02
0.00
0.20
0.15
0.10
0.05
0.00
50
40
30
20
10
0
bc
a
a
ab
c
a
c
abc
ab
a
cd
cd
bcd
d
ab
b
ab
ab
ab
a
ab
a
b
b
b
b
b
b
ab
ab
a
ab
ab
a
b
a
abc
ab
bc
c
bc
bc
field
one drip
two drips
S
F
H
P

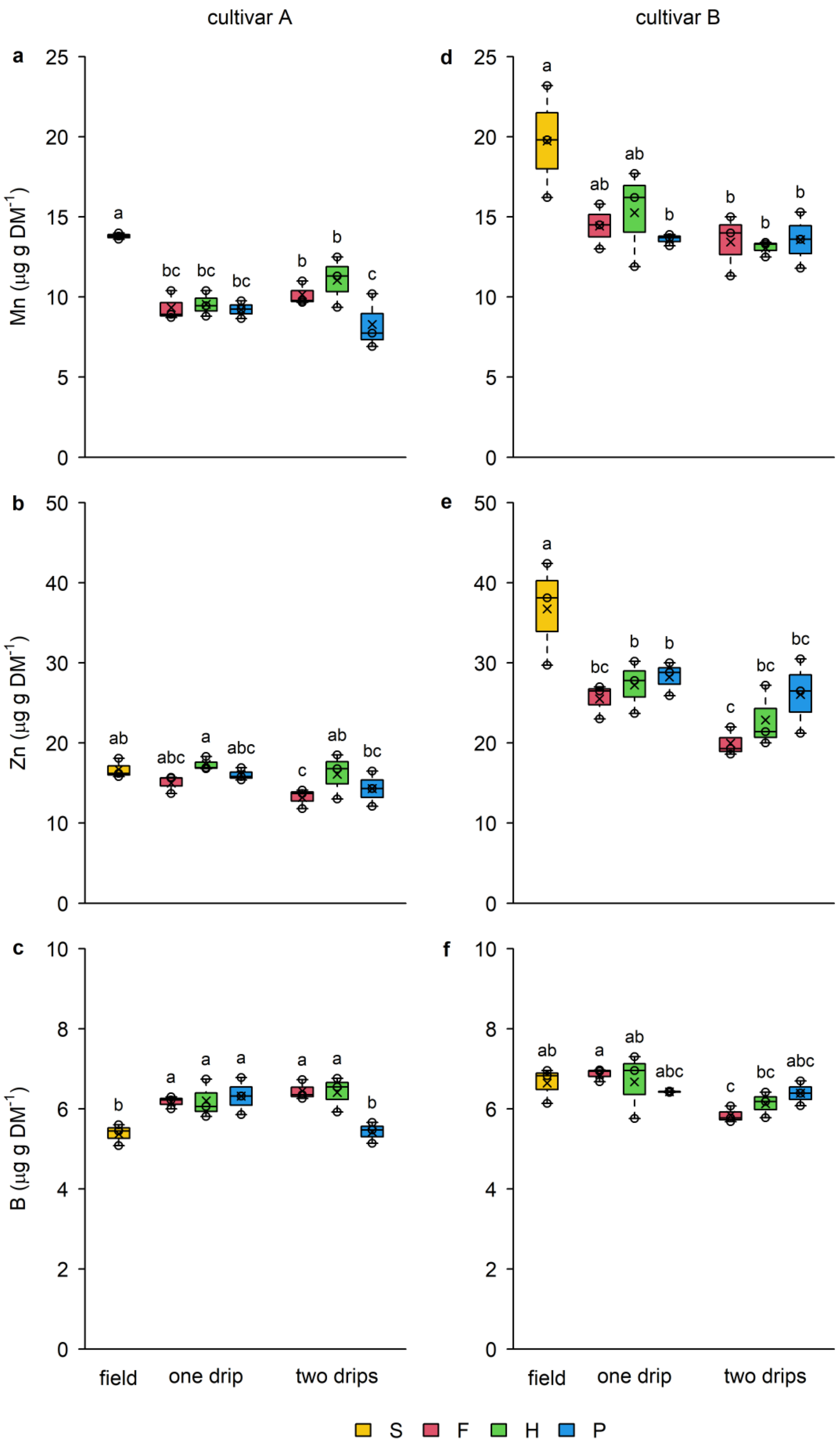
cultivar A
cultivar B
a
b
c
d
e
f
Mn (µg g DM-1)
Zn (µg g DM-1)
B (µg g DM-1)
field
one drip
two drips
S
F
H
P

**Supplementary Figure 1**

**Remaining tuber mineral composition for two tested cultivars produced in field and various hydroponic setups, as an extension of the data presented in Figure 3.**

Content of three various nutrients (minerals) measured in dry matter is presented on each single page separately for two distinct potato cultivars: celandine (a,b,c; cultivar A) and numbered cultivar (d,e,f; cultivar B). The names of the nine respective nutrients are given in the Y-axis captions. Production methods included field control in soil (S) as well as one drip and two drip hydroponic system (see Methods section for details) in three wood fiber substrates: Florentaise (F), Hunton (H) and Pindstrup (P); *n* represents the sample size, and different lower-case letters on the box plots represent significant differences between the treatments at 5% significance level. The vertical lines on the box plots indicate variability outside the upper and lower quartiles, and any point outside those lines is considered an outlier. The symbol × on the boxes indicates the sample mean; the small circles represent individual data points.

***

**Supplementary Table 1. Tuber defects (deformations, cracks, greening) and diseases (internal brown spot disease, dry rot disease, soft rot disease) observed in hydroponic production system and field control.**

| Substrate[a] | Cultivar[b] | Irrigation[c] | Deformation (%) | Cracks (%) | Greening (%) | Dry rot (%) | Brown spot (%) | Soft rot (%) |
|---|---|---|---|---|---|---|---|---|
| F | A | T | 13.8 a | 2.1 ab | 1.6 a | 0.3 a | 0 b | 0 b |
| F | A | TR | 4.5 abc | 0 b | 1.7 a | 0 b | 0 b | 0 b |
| F | B | T | 6.0 abc | 0 b | 0 b | 0 b | 0 b | 13.6 a |
| F | B | TR | 0.6 c | 0 b | 0 b | 0 b | 0 b | 9.4 ab |
| H | A | T | 0.2 c | 0 b | 0.3 ab | 0 b | 0 b | 0 b |
| H | A | TR | 12.7 ab | 4.5 a | 0.3 ab | 0 b | 0 b | 0 b |
| H | B | T | 6.3 abc | 2 ab | 0 b | 0 b | 0 b | 9.5 ab |
| H | B | TR | 6.3 abc | 0 b | 0 b | 0 b | 0 b | 3.3 ab |
| P | A | T | 3.9 bc | 1.3 ab | 0 b | 0 b | 0 b | 0 b |
| P | A | TR | 4.3 bc | 0 b | 0.6 ab | 0 b | 0.67 a | 0 b |
| P | B | T | 7.5 abc | 0 b | 0 b | 0 b | 0 b | 2.2 ab |
| P | B | TR | 5.9 abc | 0 b | 0 b | 0 b | 0 b | 1.4 ab |
| S | A | P | 0 d | 0 b | 0 b | 0 b | 0 b | 0 b |
| S | B | P | 1.5 c | 0 b | 0 b | 0 b | 0 b | 0 b |

[a] three wood fiber substrates: Florentaise (F). Hunton (H) and Pindstrup (P), and field control in soil (S)

[b] two distinct potato cultivars: celandine (A) and numbered cultivar (B)

[c] one drip and two drip hydroponic system deployed in only tuber zone (T) and in both tuber- and root zone (TR). Different lower-case letters represent significant differences between the treatments at 5% significance level.
